# A Eulogy to the Late Professor Luc Calliauw

**DOI:** 10.21315/mjms2022.29.1.14

**Published:** 2022-02-23

**Authors:** Nasser Abdul Wahab, Sharon Casilda Theophilus

**Affiliations:** 1 Neurosurgery Department, Hospital Putrajaya, Putrajaya, Malaysia; 2 Neurosurgery Department, Hospital Sultanah Aminah, Johor, Malaysia

**Figure:**
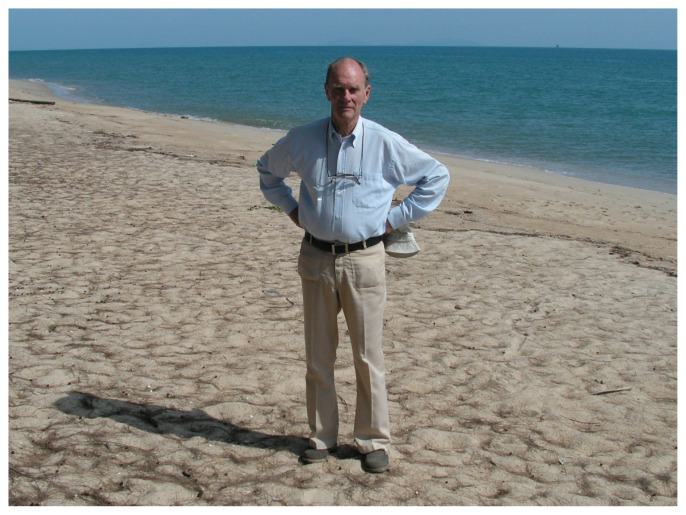


The impact of the late Professor Luc Calliauw during the early days of neurosurgical resident training in Hospital Universiti Sains Malaysia: the ‘Calliauw-isation’ of neurosurgery knowledge and how it influenced the generations of neurosurgeons who graduated from Universiti Sains Malaysia.

Professor Luc Calliauw passed away peacefully on 1 December 2021 surrounded by his children after a very long and fascinating life. He was 93 years old.

Professor Luc Calliauw was born in 1928 in Bruges, Belgium and studied medicine at Ghent University, Belgium. He was trained by Professor Verbiest in Utrecht, Holland where he was certified as a neurosurgeon in 1960 and obtained a PhD in 1968. He was involved in the training of Malaysian neurosurgeons and was a Visiting Professor to the Master of Surgery (Neurosurgery) Universiti Sains Malaysia training programme for numerous years and an Honorary Member of the Neurosurgical Association of Malaysia.

We started our postgraduate neurosurgery studies in 2002. It was the beginning of two plus four years of intense training, studies and work at the same time. A gruelling task, but because of our determination, we soldiered on. A year later we completed our part 1 foundations and entered the next stage of training, the neurosurgical residency period. We were excited upon entering the next stage because apart from more hands- on surgical training, we would be introduced to our honorary lecturer, the late Professor Luc Calliauw.

He was our mentor (Professor Jafri Malin Abdullah)’s mentor, and we heard many stories about him beforehand. He was described as a very strict, disciplined and stern person to work with, and no less than perfection was demanded at all times! Listening to these stories from our professor, made us nervous and intrigued at the same time. So, it made perfect sense when we first attended his lecture that we were on time. Papers and pens ready, and presentations of case discussions prepared beforehand, we sat up straight to attention.

At the end of the table, an elderly man was sitting, light blue shirt, white pants, brown comfortable shoes. He looked relaxed, with blue eyes staring intently at each and every one of us. As he was sitting, he was holding his glasses, nibbling at the ear tip. His hands were slender and I bet they would be as steady as a rock! He carried with him years of experience and wisdom in neurosurgery.

When he spoke, his voice was clear, sharp and straight to the point. He encouraged us to give our opinion, argue the point, and discuss and see the disease from another point of management. He was very engaging, encouraging and we truly enjoyed our sessions. We looked forward to all the following sessions as they opened our eyes and minds to new information and opinions.

Over the years, Professor Luc Calliauw came to teach every 6 months. More of us would attend his lectures as the batches of trainee neurosurgeons passed their foundation exams. In one of his visits, we spent the day travelling to a neighbouring state for a leisure visit. We thoroughly enjoyed ourselves and Professor Luc Calliauw really liked the sandy beaches of Kuala Terengganu. At that moment, he was not our professor, but an adorable elder of the family. Definitely not the fierce and stern person as described by Professor Jafri Malin Abdullah!

After numerous years, age had definitely made his travelling more difficult, so he stopped paying us visits. Our memories of him still live on with us, however, and his knowledge will forever be part of us. We were not as lucky as our Professor Jafri Malin Abdullah who had learned from him for years, but the short time he had with us was nonetheless very precious indeed.

Thank you, Professor Luc Calliauw, from all of us, the Master of Surgery (Neurosurgery) graduates at Universiti Sains Malaysia.

Rest in peace. We are eternally indebted to you.

The mediocre teacher tells. The good teacher explains. The superior teacher demonstrates. The great teacher inspires - William Arthur Ward

A teacher affects eternity; he can never tell where his influence stops - Henry Brooks Adams

